# A Literature Review and Management Approach for Severe Skin Toxicity Induced by Enfortumab Vedotin Through Sequential Adaptation and Combination With Immune Checkpoint Inhibitors

**DOI:** 10.7759/cureus.89678

**Published:** 2025-08-09

**Authors:** Yohei Iimura, Seiichiro Kuroda, Sachie Kaichi, Kisumi Yomoda, Fusako Niimi, Soh Mee Park, Sayuri Takahashi

**Affiliations:** 1 Department of Pharmacy, The Institute of Medical Science, The University of Tokyo, Tokyo, JPN; 2 Department of Urology, The Institute of Medical Science, The University of Tokyo, Tokyo, JPN; 3 Department of Pharmacy, Seoul National University Bundang Hospital, Seongnam-si, KOR

**Keywords:** enfortumab vedotin, immune checkpoint inhibitor, management, oncodermatology, skin toxicity

## Abstract

In patients with advanced urothelial carcinoma who have progressed after platinum-based chemotherapy, enfortumab vedotin (EV) improves overall survival compared to standard chemotherapy. Additionally, for treatment-naïve patients with locally advanced or metastatic urothelial carcinoma, the combination of pembrolizumab and EV demonstrates superior efficacy over platinum-based chemotherapy. Hence, EV becomes a standard treatment option. Although EV monotherapy is generally well tolerated, with severe skin toxicities occurring in some cases, higher rates have been reported when combined with immune checkpoint inhibitors (ICIs). Severe EV-induced skin toxicities have reportedly occurred. Management of these toxicities remains challenging because of inconsistent recommendations and varied responses to therapies. Hence, to determine the difference in the frequency and severity of skin toxicity induced by EV based on whether ICIs were used concomitantly with or before EV therapy, we searched PubMed, Medical Online, and Cochrane Library for articles published from January 2012 to March 2025. We included clinical trials and cohort studies. To identify data on severe skin toxicity, data were extracted with a focus on grade ≥3 skin toxicities. As a result, a total of 644 articles were identified through the literature search. Of these, 11 publications were included based on predefined eligibility criteria for the literature review. The frequency of EV-related grade ≥3 skin toxicity was higher in patients treated with EV in combination with ICIs or following prior ICI therapy, compared to EV monotherapy. Heterogeneity among studies may involve differences in the duration and method of assessment of adverse events. Specific management is needed because EV-induced skin toxicity can be more severe during concomitant or sequential ICI indications than monotherapy of EV. An inflammatory infiltrate composed of CD4+ and CD8+ T cells was identified as a contributing factor to EV-associated skin toxicity based on histopathological data from prior studies, suggesting that it shares pathologic features with ICI-associated skin toxicity. Allergic reactions were considered a contributing factor to skin toxicity induced by the combination of EV and ICI, as supported by both clinical and histopathological findings. Clinically, affected patients often presented with erythematous, pruritic rashes resembling drug eruptions, while histopathological analysis revealed features such as spongiosis, perivascular lymphocytic infiltrates with eosinophils, and interface dermatitis, consistent with hypersensitivity reactions. Antihistamines and topical steroids have anti-inflammatory effects on the above mechanisms and can be useful for EV-related skin toxicities. Therefore, prophylaxis with antihistamines and early steroid intervention at the onset of skin toxicity are needed.

## Introduction and background

In cases of metastatic bladder cancer, the five-year relative survival rate is estimated to be around 6% [1,2]. Accounting for more than 90% of all diagnoses, urothelial carcinoma is the most frequently occurring type of bladder cancer [3].

Enfortumab vedotin (EV) improves survival in platinum-refractory urothelial cancer, while EV plus pembrolizumab outperforms platinum-based chemotherapy in untreated advanced cases [4-6]. While EV is generally well tolerated, dermatologic toxicities are among the most common adverse events, with an incidence of approximately 40% [4-6], necessitating specific management strategies. Several reports on EV-induced skin toxicities have been documented [7-13]. The incidence of maculopapular rash in patients on EV monotherapy is around 25% for all grades, of which 1.4% were grade ≥3 [14,15]. Most dermatologic adverse events related to EV monotherapy, such as maculopapular rash and pruritus, are typically mild (grade ≤1) [14]. Although rare, cases of Stevens-Johnson syndrome and toxic epidermal necrolysis have been reported [16]. Further, a retrospective cohort study suggested that EV-induced skin toxicity is frequently observed after treatment with immune checkpoint inhibitors (ICIs) or when used in combination with them [17]. Notably, the number of pertinent studies has been on the rise in recent years.

Regarding the mechanism of EV-induced cutaneous toxicity, EV is an antibody-drug conjugate that targets Nectin-4, a transmembrane cell adhesion molecule highly expressed in urothelial carcinoma and other epithelial malignancies [18]. It consists of a fully human monoclonal antibody directed against Nectin-4 linked to monomethyl auristatin E (MMAE), a potent microtubule-disrupting cytotoxic agent [18,19]. On-target, off-tumor effects via Nectin-4 expression in skin Nectin-4 is not exclusively expressed in tumor cells [20,21]. It is also physiologically present in normal epithelial tissues, particularly in the skin, including keratinocytes, sweat glands, and hair follicles [20,21]. The binding of EV to Nectin-4-expressing skin cells may lead to the intracellular delivery of MMAE, resulting in apoptosis and damage to skin structures [22]. Nectin-4 plays a role in adherens junctions and epithelial cohesion [21]. Its blockade and internalization may impair intercellular adhesion, compromising skin barrier function and contributing to dermatitis-like reactions [23,24]. MMAE, the cytotoxic payload, inhibits tubulin polymerization, causing G2/M cell cycle arrest and apoptosis [25]. In rapidly renewing epithelial tissues like the skin, this can lead to keratinocyte dysfunction, delayed wound healing, and increased susceptibility to mechanical damage and inflammation [25].

Preventive measures include barrier agents and sunscreen [26-28]. Grade 1-2 toxicity is managed with topical treatments and may require EV dose adjustment. Grade ≥3 toxicity requires systemic steroids and EV interruption or reduction [26-28]. However, management recommendations are inconsistent, and the potency of topical steroids varies across recommendations [26,27]. Especially, whether or not to recommend high-potency topical steroids for the development of grade 1 skin toxicities depends on the guideline [26-28]. Given the expanding clinical use of EV combined with ICIs and the observed increase in dermatologic toxicities, there is a critical need to clarify optimal management strategies for severe skin adverse events.

## Review

Materials and methods

**Figure 1:**
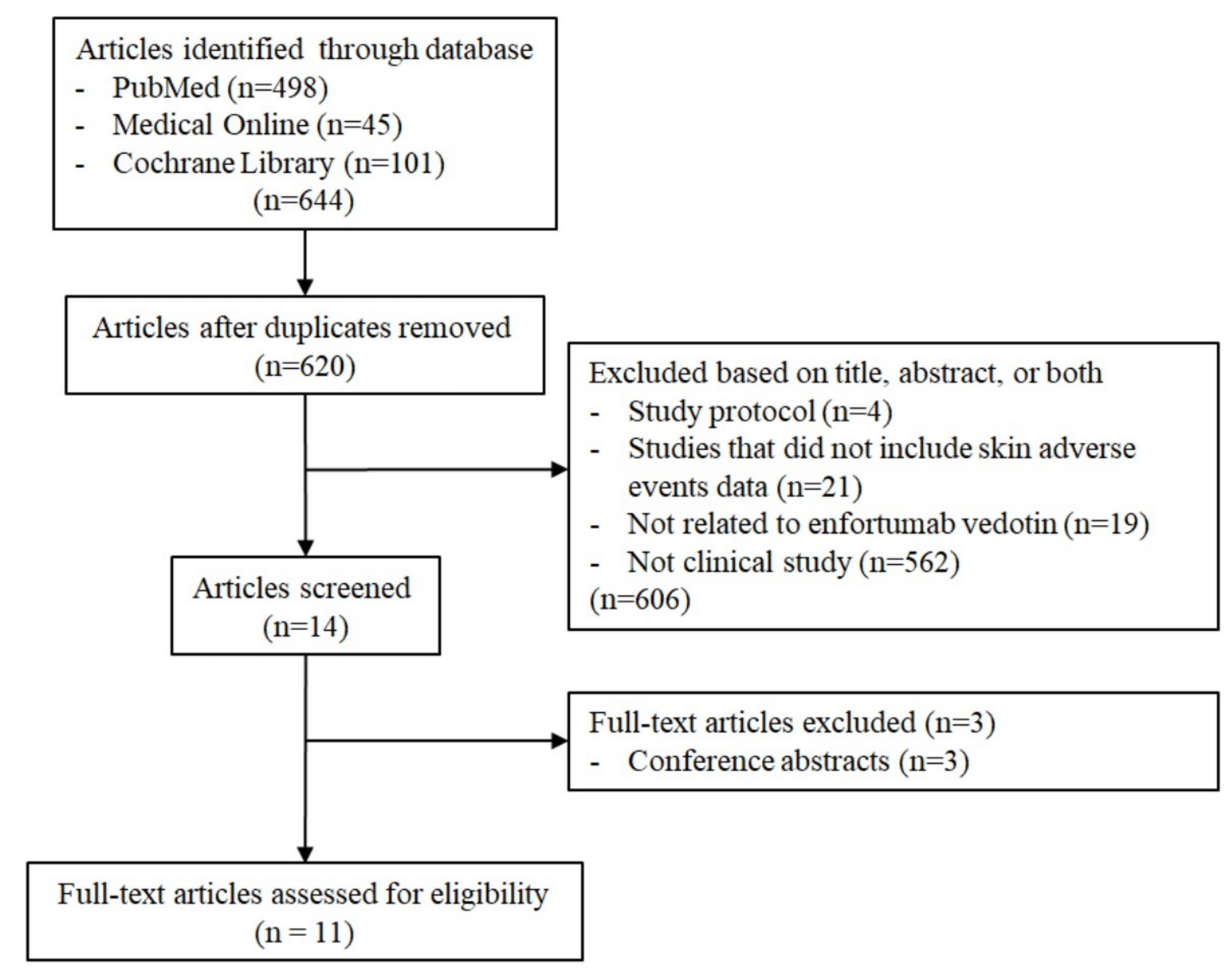
Study selection/screening process

A total of 644 articles were identified by conducting a search on PubMed, Medical Online, and Cochrane Library for articles published from January 2012 to March 2025. During the review process, all literature was extracted only from English-language articles by one reviewer. This is a narrative review, and the inclusion of studies was based on relevance rather than strict quality criteria. We focused on clinical trials and cohort studies reporting grade ≥3 cutaneous toxicities. EV, immune check point inhibitor, dermatology, and skin were used as keywords to search the database. Of these, 606 publications were excluded after reviewing the titles and abstracts because they were either irrelevant to the research topic. In addition, three articles were excluded after a thorough review because of the absence of grade≥ 3 skin toxicity events or insufficient reporting detail. Figure 1 shows the literature screening process. The methodology and reporting of this review adhere to the Preferred Reporting Items for Systematic Reviews and Meta-Analyses 2020 statement. This review did not include quality assessment tools like GRADE or Cochrane risk-of-bias.

Literature review

In all, 11 articles with skin toxicity profile data were extracted (Table 1). This summary is based on descriptive comparisons across studies, given the heterogeneity of data. Combination with ICIs consistently increased the frequency of maculopapular rash and pruritus. Notably, a marked increase in the incidence of grade ≥3 maculopapular rash was observed (descriptive results based on pooled frequencies).

**Table 1:** Difference in the incidence of skin toxicity between enfortumab vedotin monotherapy and enfortumab vedotin combined with immune checkpoint inhibitor in patients with advanced urothelial cancer Severity was evaluated using CTCAE version 5.0 (National Cancer Institute); ^*^96.8% of patients were administered immune checkpoint inhibitors as pretreatment [29]; ^**^Detailed data by type of skin toxicity (maculopapular rash, pruritus, or alopecia) were not available. All values were taken directly CTCAE: Common Terminology Criteria for Adverse Events; NA: not available Source: [4-6,14,15,17,28-32]

Therapy	Enfortumab vedotin monotherapy		Enfortumab vedotin combined with immune checkpoint inhibitor		Enfortumab vedotin after immune checkpoint inhibitor	
Severity	Any grade	Grade ≥3	Any grade	Grade ≥3	Any grade	Grade ≥3
Maculopapular rash (%)	25-28.8	1.4	32.7-68	7.7-15	16.2-30	3.2-8
Pruritus (%)	26-37	1.4	33.3-41	1.1-3.9	4.8^*^-32.4	0-3
Alopecia (%)	35.6-37.5	0	33.2-48.9	0	45.6-51	0
Skin toxicity^**^	NA	14.	NA	NA	47	12.0-44

Discussion

Based on our literature review, combined and sequential adaptation of ICIs can increase the frequency and severity of EV-induced skin toxicity (Table 1). The incidence of grade≥ 3 skin toxicity was more frequent when EV was administered after ICI. In cases where EV was administered after ICIs, any grade skin lesions occurred in 47% of the patients and grade≥ 3 in 12%-44% [17,32]. Because this variability in the data can be related to the evaluation period of each study, we should focus on the most frequently expressed values.

EV-induced skin toxicity occurs relatively early after administration [22,33] within the first few weeks; therefore, prevention and prompt response at onset are necessary. Nevertheless, data regarding the onset timing of EV-induced skin toxicity remain insufficient due to the limited sample size, underscoring the necessity for further research.

**Figure 2:**
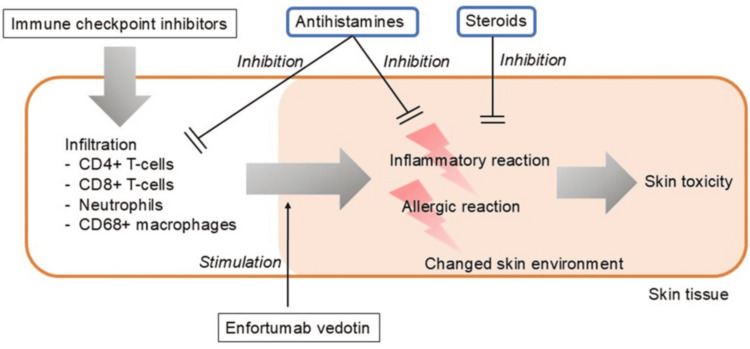
Possible mechanism of enfortumab vedotin-related severe skin toxicity after administration of immune checkpoint inhibitors (suspected mechanism) Immune checkpoint inhibitors alter the skin environment by promoting infiltration of CD4+ and CD8+ T-cells, neutrophils, and CD68+ macrophages [9]. Enfortumab vedotin triggers inflammatory and allergic reactions [54,55]. Antihistamines may help stabilize the skin environment and mitigate these reactions, while steroids suppress inflammatory and allergic reactions [49,50]. The limited number of cases constrains the robustness of the available data, thereby necessitating further comprehensive research Image credit: This is an original image created by the author Yohei Iimura

This may be attributed to immune modulation following ICI therapy, which alters the cutaneous inflammatory environment. Infiltration of CD4+ and CD8+ T-cells, neutrophils, and CD68+ macrophages leads to a specific environment conducive to severe inflammatory and allergic reactions [9]. Inhibition of Nectin-4 has been reported as a potential cause of EV-induced skin toxicity [22]. Since Nectin-4 is also present in normal skin and adnexal structures [34], EV, a Nectin-4 monoclonal antibody, can cause tissue damage in normal skin [22]. Another possible mechanism of action is the direct cytotoxic effect of MMAE [33]. This hypothesis is based on histopathological findings [33]. Cytotoxic CD8-positive lymphocyte infiltration has also been reported to be a cause of ICI-induced skin toxicity [35,36], and concomitant therapy with ICIs can exacerbate this condition [37]. Immunohistology studies have shown predominantly CD3/CD4-positive [36,38-41], mixed CD4/CD8 [42], or marked cytotoxic CD8-positive lymphocyte infiltration [35,36]. Skin toxicity tends to occur relatively early, typically within a few weeks after administration [39,43-48]. Prior ICI therapy may alter the immune environment, increasing the susceptibility to EV-induced skin toxicity, even in the absence of ICI-related skin reactions. Further studies are needed to elucidate how prior ICI exposure modulates the cutaneous immune environment to predispose to EV toxicity. The above mechanisms possibly induce a common inflammatory response. Steroids target both the inflammatory infiltrate and possible allergic components [49,50]. Steroids are recommended for treatment based on consensus recommendations and clinical practice patterns [26-28]. Since allergies are suggested to be a risk factor for the development of EV+ICI-induced skin toxicity [28], allergic reaction measures, such as antihistamines, may be effective. The prophylactic use of antihistamines remains an unproven but promising strategy requiring prospective validation. Since the development of skin toxicity may be correlated with anticancer effects of EV [51] and ICIs [52,53], appropriate management is important. However, this recommendation is not based on the results of clinical trials. Prospective trials to confirm the efficacy of prophylactic strategies and refine dose adjustment protocols are needed (Figure 2).

Possible management strategies

Several recommendations (expert opinions and clinical consensus statements) for managing EV-induced skin toxicity, including combination therapy with ICIs, have been reported [26-28]. An approach involving the use of antihistamines as prophylaxis, which differs from previous recommendations, may be recommended. Current management strategies, informed by clinical experience, include the use of antihistamines for the treatment of grade 1 dermatologic adverse events [26-28]. However, the evidence base for antihistamine prophylaxis is hypothesis-generating. As outlined in Table 2, treatment recommendations include moderate-potency topical corticosteroids for grade 1 skin toxicity as needed, high-potency topical corticosteroids for grade 2, and systemic corticosteroids for grade ≥3 toxicities (based on clinical experience). Given the potential risks associated with high-potency topical corticosteroids in the management of mild cutaneous toxicities [56], the use of moderate-potency agents is recommended based on clinical safety considerations.

**Table 2:** Possible recommendation for skin toxicity induced by combined therapy of EV and ICIs Grade should be evaluated by CTCAE version 5.0 CTCAE: Common Terminology Criteria for Adverse Events; EV: enfortumab vedotin; ICIs: immune checkpoint inhibitors; SJS: Stevens-Johnson syndrome; TEN: toxic epidermal necrolysis

Possible management recommendation	
Prevention [26,57,58]	
Moisturizing	
Sunscreen	
Avoidance of traumatic and chemical skin damage	
Administration of antihistamines (p.o. or d.i.v.)	
Skin cleansing	
Treatment	
Grade 1 [14,26,27,30,57]	Maintenance dose of EV and ICIs
	Moisturizing
	Antihistamine (p.o. or d.i.v.) for pruritus
	Moderate potency topical steroid as necessary
Grade 2 [14,27,30,57]	Consider interruption of EV and ICIs until the skin toxicity improves to grade 1
	Reintroduction of EV at the same dose or consider reducing it by one level
	High-potency topical steroids
	Emollients
	Antihistamine (p.o. or d.i.v.) for pruritus
	Topical antibiotics
Grade 3 [14,27,30,59]	Interruption of EV and ICIs until the skin toxicity improves to grade 1
	Reintroduction of EV at the same dose or consider reducing it by one level
	Very high-potency topical steroids or systemic corticosteroids
	Antihistamine (p.o. or d.i.v.) for pruritus
	Topical or systemic antibiotics
	Consider a skin biopsy
	Consider consultation with a dermatologist
Grade 4 (SJS or TEN)	Systemic corticosteroids
	Permanent discontinuation of EV

This study has a limitation. While the effectiveness of antihistamines as a premedication is suggested, further studies with larger sample sizes and controlled conditions are needed to establish their role in preventing EV+ICI-induced skin toxicity. Prospective trials assessing the efficacy of antihistamines, dose reduction strategies, and specific steroid regimens could help refine management recommendations and improve patient outcomes. Current evidence is preliminary, and antihistamines should not yet be considered standard prophylaxis. Because existing evidence is insufficient to guide optimal management, future prospective studies are urgently needed.

## Conclusions

Existing recommendations may not be sufficient to effectively manage skin toxicity in patients receiving both EV and ICIs. The classification of topical steroid strengths according to the severity of skin toxicity and prophylactic administration of antihistamines has been considered, although further validation is needed. Inadvertent use of high-potent topical steroids should be avoided to avoid steroid-induced side effects. Prophylactic use of antihistamines is a potential strategy worth further investigation. As limitations, possible publication bias, variability in skin toxicity reporting standards, and the narrative nature of the review that limits generalizability should be considered.
